# Supplementary material for: Assessing reactive violence using Immersive Virtual Reality
Source: PLoS One. 2022 May 6;17(5):e0268191. doi: 10.1371/journal.pone.0268191 (PMC9075670; doi:10.1371/journal.pone.0268191)
Supplement: Attachment — Submitted filename: Response_to_reviewers_31.01.2022.docx [file pone.0268191.s001.docx]

PONE-D-21-27929

Assessing reactive physical violence using Immersive Virtual Reality

PLOS ONE

**Response to Reviewers:**

Reviewers' comments:

*Reviewer #1: This manuscript described a novel IVR task, designed to assess aggression, and preliminary findings on its validity. Although I do concur with the authors that IVR provides completely new possibilities to assess (and treat) aggression, the manuscript requires substantial revisions before publication can be considered. I would love to see a revised version. Please see detailed suggestions and comments below.*

Thank you very much for your comments – please see below, where we have responded and changed the paper accordingly. Our responses are highlighted in yellow and changes occur as track changes in the revised manuscript.

Introduction
*In general, the introduction needs to be revised and the authors must make sure to increase clarity in conceptualizations in relation to aggression. As it stands now, the authors shortly mention key terms that are used very broad but would require more context. Please make sure to be specific and not overgeneralize, since this may lead to crucial misinterpretations. There are several, recent references which could be used to improve the introduction. For instance, see Billen et al., 2019; Berlin et al., 2021; Martens et al., 2019.*

Thank you very much for your comment. We have now added some references to improve the introduction and add more specific elements.

*p. 3, paragraph 1:
* Please provide the context for this statement, since it otherwise is too broad: "The prevalence of aggressive and violent acts is significant and requires attention and investigation."
* What do the authors mean by "Often dichotomous, violent acts arising from
aggression may be predatory, impulsive, reactive, or defensive and such behaviours may arise
from situational or environmental factors.". Please clarify what is meant by "dichotomous" in this respect.*

Thank you very much for this comment. * We have now provided context for the first statement.

*Regarding dichotomous, violent acts; Here we were referring to the fact that violence may also in some instances be reciprocal (i.e., reactive aggression following verbal aggression from the other person). However, we understand that this word might be confusing here and have now deleted it.

*p. 4, paragraph 1:
* This sentence comes without context, please remove or provide context: "Our novel IVR aggression task allows us to test levels of response inhibition in complex virtual social environments."*

Yes, indeed it does. We have removed it now and discuss our IVR task later in the introduction.

*p. 4, paragraph 2:
* The AQ does not specifically target only hostility, the current phrasing can be misleading and I this recommend revision.*

Yes, we have reworded this now to illustrate that it measures several aspects of aggression.

*p. 5, paragraph 2:
* CPT tests are not conceptualized as aggression measures, which is how it may be read as is written currently. It may, however, induce frustration. Please remove or revise.*

Thank you. We have revised this now throughout the paragraph to state that they might induce frustration.

*p.6, paragraph 2:
* VR is not a common intervention tool in aggression management, but is increasingly being tested as such in different forensic settings.
* As is written now, the reference to Klein Tuente (please revise to* *Klein Tuente) et al seems to refer to a similar intervention as Smeijers and Koole (VR-GAIME; which is only used as an add-on in ART trainings). These are two completely different methods. I recommend to either provide details for both, or just present shortly and as two independent methods.*

Yes, we agree with the reviewer. We have re-written this now, to make the 2 different approaches clearer as independent methods.

*p.7, paragraph 2: I strongly recommend the authors to not suggest that they will test causality, since this would require a completely different research design than what is provided in the manuscript at hand.*

This is a very good point. We have removed this now.

*p. 7-8: I recommend the authors to, clearly, write the scientific questions that are being addressed within the current study, so that this part can be compared to how the results are presented.*

Yes, we agree. We have added clear research questions to the end of this section now, related to the results in this study.

Methods
*p. 8: please provide more information on the participants*.

We have now added information about the participants educational background and the recruitment circumstances.

*p. 8: please provide internal consistency values for the STAXI-2 scales in the current study (or have I misinterpreted the values as coming from the reference?).*

We have added this now

*p. 8: I would suggest the authors to use "instrumental" instead of "forward" when referring to proactive aggression.*

We have done this as requested.

*Please provide a part describing the statistical considerations and analyses.*

Thank you very much for this comment. We have added more details about the analysis to the end of the methods section now.

*Results
p. 15: what measure in the IVR task was used for the correlational analyses? As it stands now, the reader has no possibility to interpret the values provided by the table. This interpretation could be facilitated by a part in statistical analyses in the methods section. This information is provided in the discussion, but must be clear already in the methods and results sections.*

We agree with the reviewer. We have now made this clearer by having added, as you suggested, the details of which values were used for the analysis at the end of the methods section.

*p. 16: the headline for Table 2 should refer to Table 3, and be followed by Table 3, I guess?*

We have changed this now.

*Discussion
My main concern on this part is that the authors use response time on the IVR task as a proxy for aggression in IVR. Unfortunately, I do not see any solid evidence-base for this assumption anywhere in the manuscript. I strongly recommend the authors to revise the aims of the study (p. 7-8) to better correspond to what was actually tested in the study, and rewrite the discussion with this in mind.*

We thank the reviewer for this excellent suggestion. And we have made this more clear now, showing at the end of the introduction as well as in the discussion that a key factor was that the response times to respond aggressively after provocation only (i.e., and not RTs in other conditions) were related to self-reported levels of aggressive behaviour.

*Minor comments:
* please proof-read once more, some words are missing in some sentences, misspellings occur (e.g. anti-social, pro-social, some references), and abbreviations should fist be introduce and then used consequently throughout the manuscript.*

We have carefully proof-read the manuscript and have made appropriate corrections

--------------------------------

*Reviewer #2: First of all, I want to congratulate the authors on their study. This manuscript concerns an important issue for society, using innovative and promising techniques. The possibility of finding more ecologically valid instruments is very interesting, for both empirical research and clinical practice. That is why I enjoyed reading the manuscript.*

We thank the reviewer very much for this kind comment. We are very happy that they enjoyed the paper and the research.

*I have some major and minor issues with the manuscript in its present form, however, which in my opinion need to be addressed before it is suitable for publication. These are listed below.*

We have addressed all comments and our responses are below highlighted in yellow and the changes are made using ‘track-changes’ in the manuscript

*1. In the abstract, it is not clear enough what the function is of the congruent and incongruent trials. Could this be explained in a few words?*

We have clarified the abstract and added an explanation in a few words as suggested.

*2. In the Introduction, concerning the following sentence: “Often dichotomous, violent acts arising from aggression may be predatory, impulsive, reactive, or defensive and such behaviours may arise from situational or environmental factors.” For readers who are not familiar with aggression concepts it may not be clear what ‘dichotomous’ refers to, especially since multiple terms from different models/authors are subsequently mentioned randomly. Please explain the dichotomous character. Furthermore, be aware that terms of different authors are mixed together in this sentence. Maybe it is preferable to stick to one model (for example: proactive versus reactive aggression according tot Dodge, or instrumental versus impulsive aggression). And, finally, add a reference here.*

We thank the reviewer for their suggestions. We have addressed these issues as requested by the reviewer. We now stick to one model and have added references. We have deleted the term ‘dichotomous’ which was referring to the binary/reactive nature of aggression (i.e., aggression might be reciprocal when stemming from provocation). As suggested in the 2^nd^ comment, we have also moved this to a paragraph further in the introduction text, to more clearly describe this concept only once. We also agree leaving out the ‘dichotomous’ term and moving the other information does make this much clearer indeed here.

*3. In the introduction, the general textual structure is difficult to follow and it is too lengthy. For example, in the first part of the introduction, the authors shift from prevalence to dichotomous aggression types, to models (with in between again dichotomous aggression types), to (yet again) similar aggression subtypes, followed by – out of the blue- an IVR aggression task, which is only explained at the end of the introduction. Please look into the flow of your introduction to make it more easy to read (for example: choose to use only one set of terms to describe the dichotomy between reactive and proactive aggression, and do this in one paragraph instead of spread around the entire introduction). Furthermore, shorten the introduction considerably (for example the second paragraph (GAM / SIP) could be removed entirely without compromising the key message of the introduction, and the same goes for various other parts of the introduction (the AQ/STAXI descriptions and provocation paradigms could be discussed in less detail. Also, the intervention study using VR could be removed from the introduction, since the present study is not an intervention study (therefore not relevant in this section and, thus, less distracting for the reader), but it may be interesting to mention this study in the discussion as another field of application for VR in relation to aggression).*

Thank you for this comment. We have now re-arranged the introduction to make it a clearer structure and easier to read. We have also deleted some elements. Indeed, the SIP model is not needed in detail here, so we have deleted the description of it. We have also shorted parts of the description of the STAXI, and left more on sentences focussing on its reliability. In accordance with the other reviewer, we have made the distinction between the two VR studies (and one being an intervention) also much clearer here and re-written this part.

*4. In the introduction: please first write the immersive virtual reality (IVR) and consequently in abbreviated form later (it is first mentioned in abbreviated form only in the first section at page 4)*

We have done this now, as suggested

*5. Introduction, page 7, concerning the following sentence: These issues of plausibility are addressed through sensorimotor contingency and domain design, creating a pragmatic method of measuring physical acts of aggression. Could the authors explain how this is done?*

We have added an explanation and examples here.

*6. In the final section of the introduction, be consequent in using past or present tense in describing the present study. And please do not jump to another previous study here (this closing part of the introduction should only describe the present study)*

We have now checked that it is all written in past tense and also deleted the reference to a previous study in this part.

*7. Introduction, last paragraph: is the aim of the study actually to develop an assessment? Should this not be: to determine if the (already) developed IVR-assessment is a good (valid) instrument to assess aggression?*

We agree with the reviewer here. We have corrected this now.

*8. Method: is it possible for the authors to give more information about the participants? Were these all students? Is it known whether they had problems with aggression in the past? And, if not, is this a study limitation? Should the task not better be assessed in clinical forensic groups who are known for high levels of aggression and where it is most likely to be used in the future? (this latter point is already appropriately discussed by the authors in the discussion section, however)*

As suggested by the reviewer, we have added more information about the participants. Indeed, most were students. We have also made it even more clear in the discussion that this was the case and that future studies should apply the task in a forensic setting.

*9. Method, STAXI: please be consequent in present or past tense. Can the anger control scales be explained briefly like the anger expression scales?*

We have now written in the past tense now and explained anger control.

*10. Method, the IVR task: can the authors give information of the degree of emotional expression on the avatar’s face and posture (in other words: hoe realistic is for example an aggressive or a friendly approach with respect to nonverbal features, apart from the lip synchronisation?)*

We have now explained in the manuscript that the only feature used to express emotion was in the lip movement on the avatar’s face and the sound/voice.

*11. Method: the authors describe that the participant sees a virtual arm to enact the reaction. However, the participants actually use a joystick to initiate this virtual response, which means that their physical sensorimotor perception is not congruent with the perceived visual response. Can the authors elaborate on what in their opinion this might mean for the ecological validity of the task (in the discussion section)?*

The reviewer makes a very good point here. Indeed, using more realistic physical device could increase ecological validity even more. We have added mention of this to the discussion, including the citation to a study in which a force producing device was used in IVR.

*12. Method, IVR: how is the response action operationalized? Please explain. For the results section: in the correlation and regression analysis it is not clear at all what type of variable is used here in the analyses to represent the IVR-conditions! This must be explained (in the discussion is mentioned that this concerns response time, but this needs to be clear in the previous sections as well). And it needs to be explained why the authors think that this measure is representative of actual aggression. Furthermore, nowhere in this article is described what the direction of the correlation is; only that it is significant. Thus, are shorter reaction times related to higher levels of aggression or are longer reaction times related to higher levels of aggression? And how do the authors explain this finding? (please elaborate on this in the discussion section) What is the relevance of it and is this known from literature?*

Indeed, a shorter response (i.e., quickly responding aggressively after provocation) was suggested to indicate higher levels of aggression. We have added this to the methods section now.

We have also added to the results section that the correlation was negative (i.e., the shorter the response time the higher the self-reported level of aggression). This is certainly very important, and we thank the reviewer for their comments.

*13. Method, IVR task: can the authors explain what the function is of the congruent versus non-congruent conditions?*

We have done this now, as requested. We have added a column to table 1 in which we now clearly describe each condition and what they indent to assess.

*14. Procedure: did the authors investigate if the order of the presented blocks in the IVR task was of influence on the outcomes of the aggression questionnaires (in other words: was there a priming effect of the IVR- conditions on the questionnaires – for example when the last condition elicited an aggressive state preceding the questionnaires - and is there corrected for such an effect?)*

This is a very good idea. However, we did not assess this in this study. We have added this as a point for future research in the discussion.

*15. Results, Table 2: What correlations are reported here? Pearson’s r? The RPAQ variables in the first column do not correspond with the RPAQ variables in the top row. (and please add RPAQ to the text above this table where correlations with reactive past aggression are described)*

Yes, *Pearson’s r*. This was missing and we have added now added this to the manuscript.

*16. Results: last paragraph: please add that Anti-Social 1 was the congruent condition*

Done

*17. Discussion: can the authors give an indication of the expected field of application for this tool? Do they see it as an instrument for empirical studies only or as an actual diagnostic tool in clinical practice? For example to assess the type and severity of a patient’s aggression problem, or to determine an individual’s improvement during aggression management treatment?*

We do indeed see this to be applied as a measurement tool in forensic settings. We have added this to the discussion.

*18. Discussion: A strength of the VR environment can be that it is fun to do, which might make individuals more willing to cooperate with such assessments, which is often a problem in forensic populations. The authors might stress this more.*

This is very true. We have added this point to the discussion.

*19. Discussion: Might it be a valuable addition for the future to combine the IVR task with biomarkers such as heart rate or skin conductance to even better assess aggressive responses?*

As the reviewer suggests, it might be indeed. However, we believe that there might be quite a bit of movement/noise in such measurements. But indeed, we believe that future research looking into such signals is a great idea, and we have now also added this point to the discussion.

*20. In the abstract is suggested that IVR is ethically safe. This point is not mentioned in the manuscript. Could it be explained in the discussion section how the authors see this?*

It is seen as safe in the sense that no real human is harmed but that the task involves virtual humans. We now mention this again in the discussion as requested.

*21. References: 31, Klain Tuente is spelled incorrect (this should be Klein Tuente)*

Reference corrected.

*Reviewer #3: This is an interesting study showing the potential usefulness of IVR for assessment purposes and (potentially) for treatment of aggressive people. The main problem has to do with the sample composition: the authors should move the section 'Participants' to the start of the Results section. Moreover they should give many additional details about the participants: education; occupation; any history of mental disorders or psychological treatment; any history of offenses, etc. Once they have added these variables (hoping that they have this info!!), they should make additional analyses to see whether any of these important sociodemographic features is associated to different IVR responses. Incidentally, the sample is clearly composed by university students, as shown by the young mean age. Moreover, the large majority of the participants are women (30 males out of 116 participants): the authors must comment on this very important sample feature, since a century of research on aggression has shown that aggressive behaviour, of any kind, is largely more frequent and severe in males compared to females. They should have considered this during recruitment and should have tried at least to achieve a fair gender balance in the sample composition. Therefore the over-representation of young females represents a major limitation of this study and has to be acknowledged.*

We thank the reviewer very much for their comment. And indeed, we agree. We have collected the educational status of the participants (but not their history of mental health problems) and now added this information to the participants section. The vast majority of participants were of same educational status, thus making cross educational background not possible to analyse. However, in the results section we have reported the correlation with self-reported past violence, which is the RPAQ score in table 2. We do agree strongly with the reviewer’s key point that most participants were students and female. This initial sample was used as first validation of the task, but off course future studies would need to apply and replicate the results using forensic sample. We have now provided much more acknowledge of this in the discussion of the paper.

*The potential utility of IVR for treatment purposes, including its utilization in clinical populations, has to be demonstrated and so far there is nothing which can support this claim: this has to be made clear.*

We now make it clear in the discussion that we are proposing this task as being a stepping stone to use IVR as an assessment tool (i.e., and not for treatment purpose).

*There are a few typos which have to be corrected:
- page 3: para 2, add 'how' after 'describes'.*

*Done
- page 5: line 10, drop 'this'.*

*Done
- page 6: correct 'aggreagable' in 'agreeable'.*

*Done*

*- same page, end page: add ' to 'participants'.*

*Done
- page 7: line 2, 'change'*

*Done*
