# Supplementary material for: Assessing reactive violence using Immersive Virtual Reality
Source: PLoS One. 2022 May 6;17(5):e0268191. doi: 10.1371/journal.pone.0268191 (PMC9075670; doi:10.1371/journal.pone.0268191)
Supplement: Attachment — Submitted filename: responses_to_reviewers_30.03.22.docx [file pone.0268191.s002.docx]

*Reviewer #2: In general, I think the manuscript is greatly improved compared to the first submission. The authors have addressed all of my previous considerations appropriately. I still have some minor comments, which I explain below. If these points can be adjusted by the authors, I believe the manuscript is suitable for publication in Plos One.*

Thank you very much for your comments. We have now addressed the issues you have raised in our 2^nd^ revision.

Our responses to your comments are below highlighted in blue and marked in track-changes in the manuscript.

Abstract:
- In the sentence: "In congruent trials, ..."please enter 'the' before 'participant' (or change into 'participants', plural).
- Last sentence: suggest should be suggests

We have made these corrections.

*Introduction:
The introduction has definitely been improved, compared to the first submission. It's easier to read and has a better flow to it. There are some minor points to consider:*

*- p3 the first lines don't fit well together. I suggest the authors remove the sentence 'The General aggression model... lead to aggression.", since there is no further mention of the model anywhere in the paper, and place the following section directly after the definition of aggression (which needs to be placed between '...' by the way, and a '.' needs to be removed at the end of that line).*

We have deleted the sentence now and moved the paragraph to the line before, removing the “.”.

*- p3 the last sentence in this paragraph needs to be clarified with respect to how the reader should interpret 'discrete social dynamics' and 'therefore' (I don't see how the second part of this sentence can logically be derived from the first).*

This is a helpful suggestion, thank you. We have now clarified the sentence.

“Furthermore, triggers for aggressive behaviours may involve discrete social dynamics [7] and in order to produce a none-aggressive response to another person, some individuals might activate inhibition and self-control to consciously inhibit their aggressive behavioural impulses.”

*- p4 in the section on the STAXI, the sentence "However, studies have shown mixed results", please add the subject of the studies here*

We have added this now.

“However, studies investigating the relationship between the STAXI score and observed aggressive behaviour, have shown mixed results.”

*- what is 'impression management'? Please explain*

We have explained this term now. “… impression management (IM, i.e., giving answers which are perceived to be socially acceptable/desirable),…”

*- p5, last paragraph, first line. VR has not yet been implemented as an intervention tool in clinical practice, but only in studies. Please clarify this.*

We have clarified this now.

*- p6: authors suggest that their task assesses levels of physical reactive aggression. I wonder if you can regard this as a measure of physical aggression if it occurs in a virtual situation (since the virtual environment implies that it is not physical at all!). Would it not be on the safer side to characterize this as 'reactive aggression'?*

We agree with this very helpful comment. We have now changed the term “physical reactive aggression” to “reactive aggression” in the title and throughout the paper.

*-p 7. Authors now state much clearer that they use reaction time as a proxy for aggression. However, it is still not clear to me why they assume that a faster reaction time is indicative of aggressive tendencies?*

We have clarified this issue now, suggesting that faster aggressive responses might indicate aggression as a rapid impulsive/accessible response type.

“This suggests, that the faster someone would exhibit the aggressive response after provocation, the more accessible that response option might be to that person, suggesting potential more impulsive aggressive tendencies.”

*Method:
- p 13, table 1. Why is an agrressive response to a friendly approach (pro-social 2, incongruent) regarded as a measure of inhibition? This is not an inhibitory response in my opinion... Or do authors think that it is inhibitory due to the incongruent nature of the response?*

Yes, indeed it is due to the incongruent nature of the task. We have clarified this now in the descriptive legend underneath the table and added the sentence:

“Note that we termed both responses to the incongruent trials as response inhibition due to the incongruent nature of the responses.”

*- p 14. last sentence, shorter reaction times are regarded as representing greater levels of aggression. Is this only in the Anti-social 1 condition? Please add this information here*

We now state that this is only for the anti-social condition 1

“We have operationalised that shorter reaction times in the Anti-Social 1 condition (…”

*- p15. Participants were told that they could leave at any time when uncomfortable. How many did actually leave?*

No participant reported to have felt uncomfortable and no participant left. We agree that this is an important point and snow state this fact in the manuscript.

“No participant reported that they felt uncomfortable, and no participant left.“
*Results:
- p16: almost half of the participants made errors during the IVR task. What type of errors should we think of here? What does this imply for validity of the test results with respect to reaction time? This point is not yet addressed in the discussion.*

We have now added this to the discussion that errors constitute doing the opposite response to the one required (i.e., handshake instead of aggressive response).

“Participants however, made also errors in the IVR task, which constituted doing the opposite response to the one required (e.g., they were hitting the virtual human when a handshake was required).”

We have also added that adding that few participants made more than one error and that errors were not correlated to the self-report questionnaire measures.

“Overall, a few participants (13.8% in incongruent and 19.8% in the congruent) made more than one error, and the number of errors were not correlated to the self-report measures.”

We now also mention that future modifications of the task could be to disable to opposite response on the response device.

“Errors in response will add noise to the results, but should not affect the underlying trends. To reduce this effect, future studies could disable to option to perform the opposite response on the IVR response device during the task.”

*- p 16: how do the authors explain that more mistakes were made in congruent than incongruent trials, while you would expect the opposite? Doe this finding need further elaboration in the discussion?*

We have elaborated on this in the discussion, stating that the responses in the congruent condition might have evoked a more emotional/intuitive/impulsive response after provocation in IVR. On the contrary the incongruent trials might have elicited more cognitive processing in participants, focussing on providing the opposite response rather than being emotionally affected by the provocation of the avatars.

“We found that people made more errors in the congruent condition compared to the incongruent one. This might indicate that the congruent condition was processed by participants in a more emotional manner (i.e., with an intuitive and impulsive response after the virtual human’s approach); in contrast they might have engaged a more cognitive approach in the incongruent condition, in which they were concentrating more about providing the correct response rather than acting on impulse.”

*Discussion:
- p 18, first paragraph of the discussion. Please add the direction of the relationship (thus: higher levels of self-reported aggression were correlated to lower reaction times on the IVR paradigm)*

We have added the following sentence about the direction as requested.

“higher levels of self-reported aggression were correlated to lower reaction times on the IVR paradigm.”

- *p 20, second paragraph, "Also, since IVR is usually regarded...". please check the grammar of this sentence (since and thus combined seems redundant)*

Thank you for your comment. We have corrected the grammar of this sentence

“Additionally, IVR is usually regarded as an engaging and ‘fun’ tool, thus making it potentially more attractive for participants to engage with the technology.”

*- p 20, bottom section, "This could be achieved by...", before during, after treatment -- > before, during and after treatment*

Updated as suggested.

*- p 20, last sentence 'be made and to that': remove 'and' here?*

We have changed this sentence.


*References
- p 23, remove [19] between 14 and 15*

We have removed this mistake.

Reviewer #3:

*In my previous review of the manuscript I wrote: "THE LARGE MAJORITY OF THE PARTICIPANTS ARE WOMEN (30 MALES OUT OF 116 PARTICIPANTS): THE AUTHORS MUST COMMENT ON THIS VERY IMPORTANT SAMPLE FEATURE, SINCE A CENTURY OF RESEARCH ON AGGRESSION HAS SHOWN THAT AGGRESSIVE BEHAVIOUR, OF ANY KIND, IS LARGELY MORE FREQUENT AND SEVERE IN MALES COMPARED TO FEMALES. THEY SHOULD HAVE CONSIDERED THIS DURING RECRUITMENT AND SHOULD HAVE TRIED AT LEAST TO ACHIEVE A FAIR GENDER BALANCE IN THE SAMPLE COMPOSITION. THEREFORE THE OVER-REPRESENTATION OF YOUNG FEMALES REPRESENTS A MAJOR LIMITATION OF THIS STUDY AND HAS TO BE ACKNOWLEDGED". I was expecting to see this issue clearly mentioned in the section on 'Study limitations' (please add a sub-heading for Limitations). In their rebuttal letter the authors write: "We do agree strongly with the reviewer’s key point that most participants were students and female. This initial sample was used as first validation of the task, but off course future studies would need to apply and replicate the results using forensic sample. We have now provided much more acknowledge of this in the discussion of the paper".
Since a selection bias in the sample composition is always a major threat to study validity, this issue should be clearly discussed and clarified in the Limitation section. Unfortunately, this has NOT been done, and in my opinion the paper should NOT be accepted until I see a clear, detailed para dealing with this major study limitation (selection bias in sample composition).*

Thank you very much for your comments. We have now addressed the issues you have raised in our 2^nd^ revision.

We have now included a subsection in the discussion with the subheading “Limitations”. Here we mention more clearly that participants where mostly students and female and that future studies would need to be done using more male participants and a forensic sample. We have written 2 paragraphs discussing this issue (See page 19 and 20.).

“Limitations

Our study involved mainly students, of similar age and educational background, with the majority of them being female. Physical violence has however been reported to be greater in men [2], thus future studies would need to include more male participants to further validate the IVR task. Additionally, the task would need to be tested in a forensic sample.

Specifically, future research might see the IVR task performed on a sample of clinical interest such as violent offenders. We used this sample as the first form to validate our new IVR measure. Future studies would now be needed to assess and replicate the findings using different samples as described above. Previous research has proposed and demonstrated behavioural measures to perform better in populations where the targeted variable occurs at a greater rate and magnitude [30]. Direct comparisons of sample characteristic would allow for further analysis of the relationship and constructs of aggression and executive functioning, where clinically relevant features such as psychopathy or violent tendencies may moderate the relationship between self-control and aggression. These comparisons give potential for an accepted taxonomy of anger and aggression and resolve the issues raised by Lee and DiGiuseppe [31] by better informing targets of treatment and rehabilitation.”
